# The quorum sensing peptide BlpC regulates the transcription of genes outside its associated gene cluster and impacts the growth of *Streptococcus thermophilus*

**DOI:** 10.3389/fmicb.2023.1304136

**Published:** 2024-01-08

**Authors:** Michael J. McAnulty, Giselle K. Guron, Adam M. Oest, Amanda L. Miller, John A. Renye

**Affiliations:** Dairy and Functional Foods Research Unit, Eastern Regional Research Center, Agricultural Research Service, U.S. Department of Agriculture, Wyndmoor, PA, United States

**Keywords:** bacteriocin, BLP, quorum sensing, transcriptome, growth

## Abstract

Bacteriocin production in *Streptococcus thermophilus* is regulated by cell density-dependent signaling molecules, including BlpC, which regulates transcription from within the bacteriocin-like peptide (*blp*) gene cluster. In some strains, such as *S. thermophilus* ST106, this signaling system does not function properly, and BlpC must be supplied exogenously to induce bacteriocin production. In other strains, such as *S. thermophilus* B59671, bacteriocin (thermophilin 110 in strain B59671) production occurs naturally. Here, transcriptomic analyses were used to compare global gene expression within ST106 in the presence or absence of synthetic BlpC and within B59671 to determine if BlpC regulates the expression of genes outside the *blp* cluster. Real-time semi-quantitative PCR was used to find genes differentially expressed in the absence of chromosomal *blpC* in the B59671 background. Growth curve experiments and bacteriocin activity assays were performed with knockout mutants and BlpC supplementation to identify effects on growth and bacteriocin production. In addition to the genes involved in bacteriocin production, BlpC affected the expression of several transcription regulators outside the *blp* gene cluster, including a putative YtrA-subfamily transcriptional repressor. In strain B59671, BlpC not only regulated the expression of thermophilin 110 but also suppressed the production of another bacteriocin, thermophilin 13, and induced the same YtrA-subfamily transcriptional repressor identified in ST106. Additionally, it was shown that the broad-spectrum antimicrobial activity associated with strain B59671 was due to the production of thermophilin 110, while thermophilin 13 appears to be a redundant system for suppressing intraspecies growth. BlpC production or induction negatively affected the growth of strains B59671 and ST106, revealing selective pressure to not produce bacteriocins that may explain bacteriocin production phenotype differences between *S. thermophilus* strains. This study identifies additional genes regulated by BlpC and assists in defining conditions to optimize the production of bacteriocins for applications in agriculture or human and animal health.

## Introduction

Bacteriocins are ribosomally encoded antimicrobial peptides that have the potential to act as preservatives within food or as alternatives to clinically relevant antibiotics. The lantibiotic nisin, naturally produced by strains of *Lactococcus lactis*, gained approval by the FDA for use as a food biopreservative in 1988 and remains the only bacteriocin with FDA approval in purified preparations (McAuliffe et al., 2001). However, several other food-grade lactic acid bacteria have been shown to produce broad-spectrum bacteriocins; thus, efforts continue to characterize novel bacteriocins and develop methods for optimizing their production (Perez et al., 2014; Barcenilla et al., 2022).

**Figure 1:**
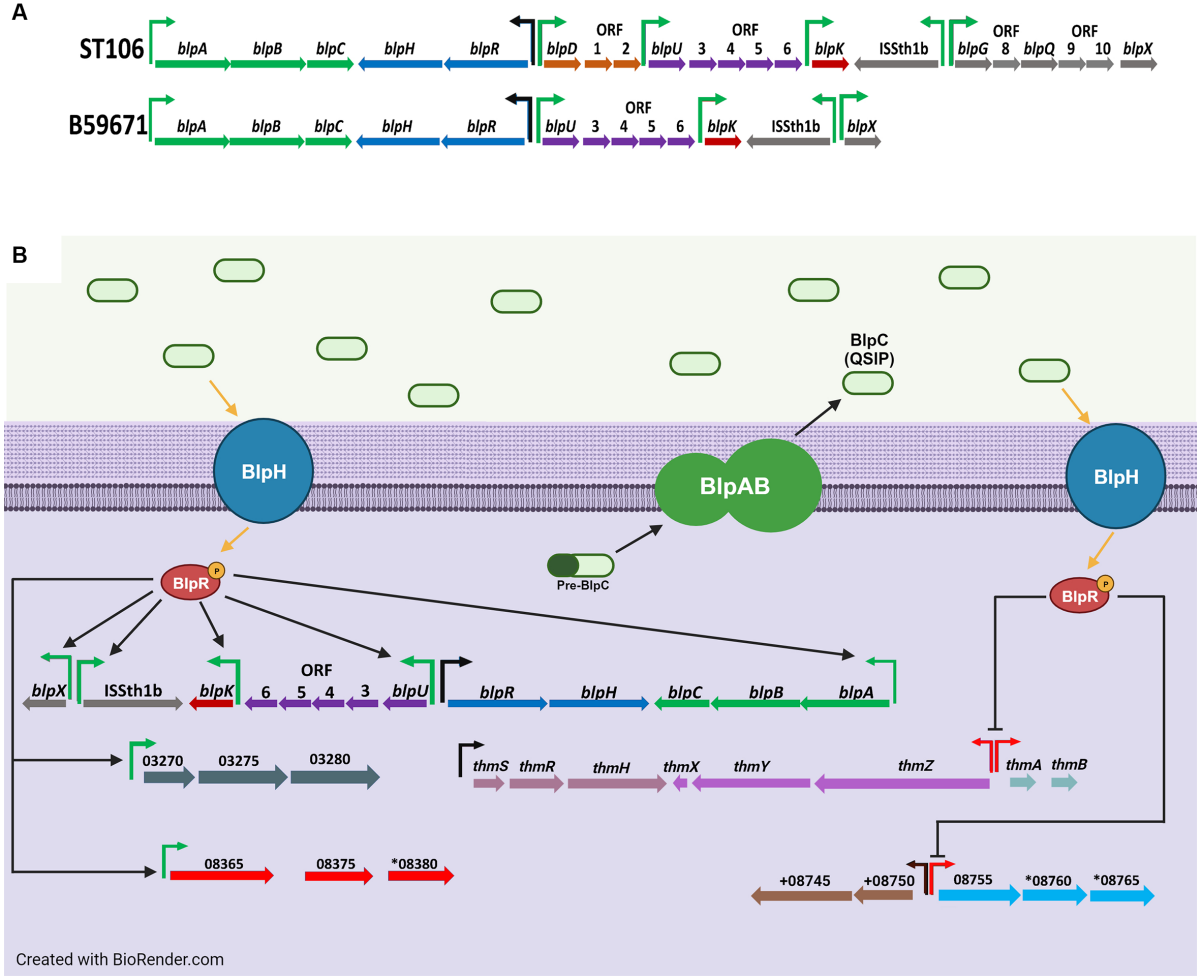
**(A)** Genetic organization of the *blp* cluster in ST106 and B59671, and **(B)** transcriptional effects from BlpC-mediated signal transduction on the *blp* cluster and other gene clusters in B59671. Putative promoters for operons in which at least one gene is significantly upregulated (green), none of the genes are significantly affected (black), and at least one gene is significantly downregulated (red) during BlpC-mediated signal transduction are shown. For simplicity, upregulation processes are on the left, and downregulation processes are on the right. Gene orientation is according to the reverse strand in panel **(A)** for visualization and according to the sense strand in panel **(B)**. Figure created with BioRender.com.

*Streptococcus thermophilus* is an industrially relevant lactic acid bacterium used in the production of yogurt and hard cheeses. Many of the bacteriocins produced from *S. thermophilus*, called thermophilins, are encoded by the bacteriocin-like peptide (*blp*) gene cluster. Strains B59671 (Gilbreth and Somkuti, 2005; Renye and Somkuti, 2013), ST106 (Somkuti and Renye, 2014; Renye et al., 2019a; Figure 1A), LMD-9 (Fontaine et al., 2007; Fontaine and Hols, 2008), ST109 (Renye et al., 2019b), and ST118 (Somkuti and Renye, 2014) all produce bacteriocins encoded within the *blp* cluster. The *blp*-encoded bacteriocins produced by ST106 and B59671 are called thermophilin 106 and thermophilin 110, respectively.

The bacteriocins encoded within the *blp* locus display activity against a wide spectrum of Gram-positive bacteria. For instance, ST109 has activity against other *S. thermophilus* strains (Fontaine et al., 2007) in addition to other Gram-positive bacteria, including the pathogens *Listeria monocytogenes* (Fontaine and Hols, 2008), *Enterobacter faecalis* (Fontaine and Hols, 2008), and *Streptococcus pyogenes* (Renye et al., 2019b). B59671 can inhibit the growth of a potential wine spoilage bacterium, *Pediococcus acidilactici* (Gilbreth and Somkuti, 2005), as well as the opportunistic pathogens *Streptococcus mutans* (Renye and Steinberg, 2021), *Cutibacterium acnes*, and *Listeria monocytogenes* (Ceruso et al., 2021). Therefore, thermophilins may have the potential to be used as food preservatives or antimicrobials for human and animal health applications.

Comparative genomics led to the discovery of the *blp* cluster in *S. thermophilus* due to its homology with the *blp* cluster of *Streptococcus pneumoniae* (de Saizieu et al., 2000). Originally, it was thought that *S. thermophilus* was unable to produce bacteriocins (Hols et al., 2005). However, it was later shown that bacteriocin production from strains LMD-9 (Fontaine et al., 2007) and ST106 (Renye et al., 2016) occurred upon the exogenous addition of a 30-mer quorum sensing induction peptide (QSIP) naturally encoded by *blpC*. When expressed, BlpC is processed into mature QSIP and transported via an ABC transporter system encoded by *blpA* and *blpB* (upper portion of Figure 1B). A histidine kinase (BlpH) and a response regulator (BlpR) form part of a signal cascade to help QSIP activate genes in the *blp* cluster, including the gene(s) encoding the bacteriocin. The overall action of the *blp* cluster causes the production of bacteriocin once a population quorum has been established.

*Streptococcus thermophilus* strains that naturally produce bacteriocins encoded within the *blp* gene cluster have also been identified, including strains B59671 and ST109 (Renye et al., 2016). In addition to genes encoding thermophilin 110, the chromosome of B59671 also has a cluster containing genes encoding thermophilin 13, another class II bacteriocin, along with a unique ABC transporter system QSIP, histidine kinase and response regulator protein, and a BlpS-type LytTR transcriptional regulator (Salini et al., 2023).

The promoter region of *blpABC* is identical between strains B59671 and ST106, and these strains display contrasting bacteriocin production phenotypes in terms of an exogenous BlpC requirement. The reason for this remains unknown. Moreover, the action of BlpC has only been studied with regard to genes within the *blp* cluster. We set out to determine the effects of BlpC on a transcriptomic level in the hopes of identifying any other genes regulated by this signaling molecule and understanding the effects of bacteriocin production on overall cell growth to potentially identify associated trade-offs.

## Materials and methods

### Bacterial strains and growth conditions

*Streptococcus thermophilus* strains ST106, B59671, and ST113 from our in-house collection were maintained in the Tryptone, Yeast Extract, Lactose (TYL) medium (Somkuti and Steinberg, 1986) at 37°C without agitation. Bacteriocin production was induced in strain ST106 by supplementing TYL with 250 ng/mL of a synthetic 30-mer peptide, corresponding to the quorum sensing induction peptide (BlpC) for the bacteriocin-like peptide (*blp*) gene cluster, at the time of inoculation (Renye et al., 2016). *Cutibacterium acnes* ATCC6919 (American Type Culture Collection, Manassas, VA, United States) was cultured in brain heart infusion (BHI) medium (Becton Dickinson Co., Franklin Lakes, NJ, United States) and grown anaerobically in a Whitley DG250 anaerobic workstation (Microbiology International, Frederick, MD, United States).

### Transcriptomic analysis

Growth of *S. thermophilus* strain ST106, with and without BlpC induction, and strain B59671 in TYL broth was monitored using a Biotek Cytation 5 multimode plate reader (Agilent, Wilmington, DE, United States). Cells were harvested in early (OD_600_ 0.15–0.25) and late (OD_600_ 0.5–0.7) exponential phases and treated with RNAlater® (Thermo Fisher, Philadelphia, PA, United States) prior to storage at −80°C. Frozen cells were sent to Igenbio Inc. (Chicago, IL, United States) for RNA Sequence (RNA-Seq) analysis. RNA was extracted, quality controlled, converted to cDNA, and sequenced using a TapeStation (Agilent), NEBNext Ultra II (Directional) RNA with QIA seq FastSelect-(5/16/23 s Total RNA-Seq; New England Biolabs, Ipswich, MA, United States), and a MiSeq (Illumina, San Diego, CA, United States) with 2 × 150 20 M paired-end reads, respectively.

The genomes of strains ST106 (Renye et al., 2019a) and B59671 (Renye et al., 2017) were completely sequenced in previous studies. Analysis of the RNA-seq data was performed using Geneious Prime software (Biomatters Inc., Boston, MA, United States) and by Igenbio Inc. using their proprietary ERGO 2.0 platform. Bowtie2 (Langmead and Salzberg, 2012) was used for aligning to reference genomes (CP031881 for ST106 and NZ_CP022547 for B59671) and DESeq2 for differential gene expression (DGE; Love et al., 2014). For strain-to-strain comparisons, reads were mapped to B59671. Read counts for each gene were normalized and then averaged across the three biological replicates. Raw reads were deposited in the NCBI Sequence Read Archive under the BioProject number PRJNA 1021576.

### Real-time semi-quantitative PCR

Cells were harvested in early (OD_600_ 0.15–0.25) and late (OD_600_ 0.5–0.7) exponential phases and treated with RNAlater® prior to storage at −80°C. RNA was isolated using TRIzol reagent (RNAwiz, Thermo Fisher) and the Direct-zol RNA Microprep Kit (Zymo Research, Irvine, CA, United States) and treated with Turbo DNase (Thermo Fisher) to remove contaminating DNA. RNA was converted to cDNA using SuperScript IV VILO reverse transcriptase (Thermo Fisher).

The cDNA libraries were diluted 1:40 in nuclease-free water and analyzed by qPCR using FastStart Essential DNA Green Master mix (Roche, Basel, Switzerland) and a LightCycler 96 (Roche). Each reaction consisted of 10 μL of 2× FastStart Essential DNA Green Master Mix, 1–2.5 μL of 4 μM of each of the forward and reverse primers (Supplementary Table 1), 2 μL of 1:40 diluted cDNA (approximately 80 pg/μL), and nuclease-free water to make for a 20 μL total volume. The reference gene used was *rlmD*, which was determined to be stable from the RNA-seq data.

The efficiency of each primer pair was determined by a calibration curve with dilutions of pooled samples containing at least four points and found to be between 85 and 100%. Melting curve analyses were used to confirm the presence of no non-specific amplification products, and pooled non-reverse-transcription negative controls were used to confirm no significant amplification was due to leftover DNA after DNase treatment or primer dimers. Confidence intervals and *p* values were calculated in Excel based on efficiency-weighted C_q_ values according to the common base method (Ganger et al., 2017).

### Gene knockouts

DNA fragments for use in Gibson Assembly were generated using PCR that used Q5 Hot Start High-Fidelity DNA Polymerase (New England Biolabs), according to the manufacturer’s instructions. The PCR was run with the following steps: 98°C for 30s, 30 cycles of 98°C for 10 s, 50°C for 30 s, 72°C for 1 min, and a final extension of 72°C for 2 min. The template was either chromosomal DNA extracted using PrepMan® Ultra Sample Preparation Reagent (Thermo Fisher) or the pKS1 plasmid (Shatalin and Neyfakh, 2005) that was obtained using the QIAprep Spin Miniprep Kit (Qiagen, Hilden, Germany) on an overnight culture of *Escherichia coli* DH5α growing in Brain Heart Infusion Media (Research Products International, Mount Prospect, IL, United States) with 150 μg/mL of kanamycin for the fragment containing the kanamycin resistance gene *kanR*. DNA fragments were purified using a QIAquick PCR Purification Kit (Qiagen). The Gibson assembly was performed using the Gibson Assembly Master Mix Kit (New England Biolabs) according to the manufacturer’s instructions, using 83 ng of each fragment.

Overnight cultures were refreshed 1:100 in 1 mL of M17-lactose and incubated at 37°C. Once each culture reached an OD_600_ of 0.05, 1.8 μg/mL of ComS 17–24 (competence-inducing peptide; Gardan et al., 2013; Lingeswaran et al., 2020) and approximately 250 ng DNA (*kanR* flanked by 1.1 and 1.2 kb of the flanking segments of the gene to be knocked out) were added. Cultures were incubated another 2 h before plating 100 μL of aliquots on TYL with 150 μg/mL of kanamycin. Mutations were verified by culture PCR.

### Supernatant bacteriocin activity

Bacteriocin activity was measured using a well-diffusion assay (Renye et al., 2016). Briefly, supernatants (~35 μL) were loaded in pre-cast wells in agar medium seeded with 1% (v/v) of an overnight culture of *S. thermophilus* or *C. acnes* 6919. The plate was kept at 4°C overnight and then incubated at 37°C until growth was visible. *Cutibacterium acnes* dishes were incubated anaerobically in a Whitley DG250 Workstation (Microbiology International, Frederick, MD, United States) for 72 h. Antimicrobial activity was determined by the presence of a zone of inhibition around wells.

### Growth curves and cell viability

Cells from overnight cultures were washed by centrifuging at 10,000 × *g* for 2 min at 4°C and resuspending in two volumes of TYL. After taking OD_600_ readings of these inocula, calculated volumes of the inocula were then put in 5 mL of TYL with or without BlpC for a starting OD_600_ of 0.008. A measure of 200 μL of aliquots was placed in a 96-well plate, incubated at 37°C in a Biotek Cytation5 multimode reader, and after 1 h time increments, the plate was shaken for 5 s orbitally and OD_600_ measured to assess biomass.

Cell viability was determined after 24 h of growth using a method modified from Chen et al. (2003). Briefly, triplicates for each biological replicate of 5 μL droplets of cultures serially diluted in 0.1% peptone water were dropped onto TYL agar, and plates were incubated at 37°C for 48 h. Colonies were counted and reported as aerobic plate counts (APCs) and colony-forming units (CFUs) per mL.

### Statistical analysis

Unless mentioned elsewhere, analyses were performed using Microsoft Excel, and ANOVA statistical analyses were performed using R 4.1.1 (R Core Team, 2021). Graphs were made using ggplot2 in the R interface (Wickham, 2016). Signal peptides were predicted using SignalP 6.0 (Teufel et al., 2022).

## Results

### Effects of exogenous BlpC on the transcriptome of ST106

**Figure 2:**
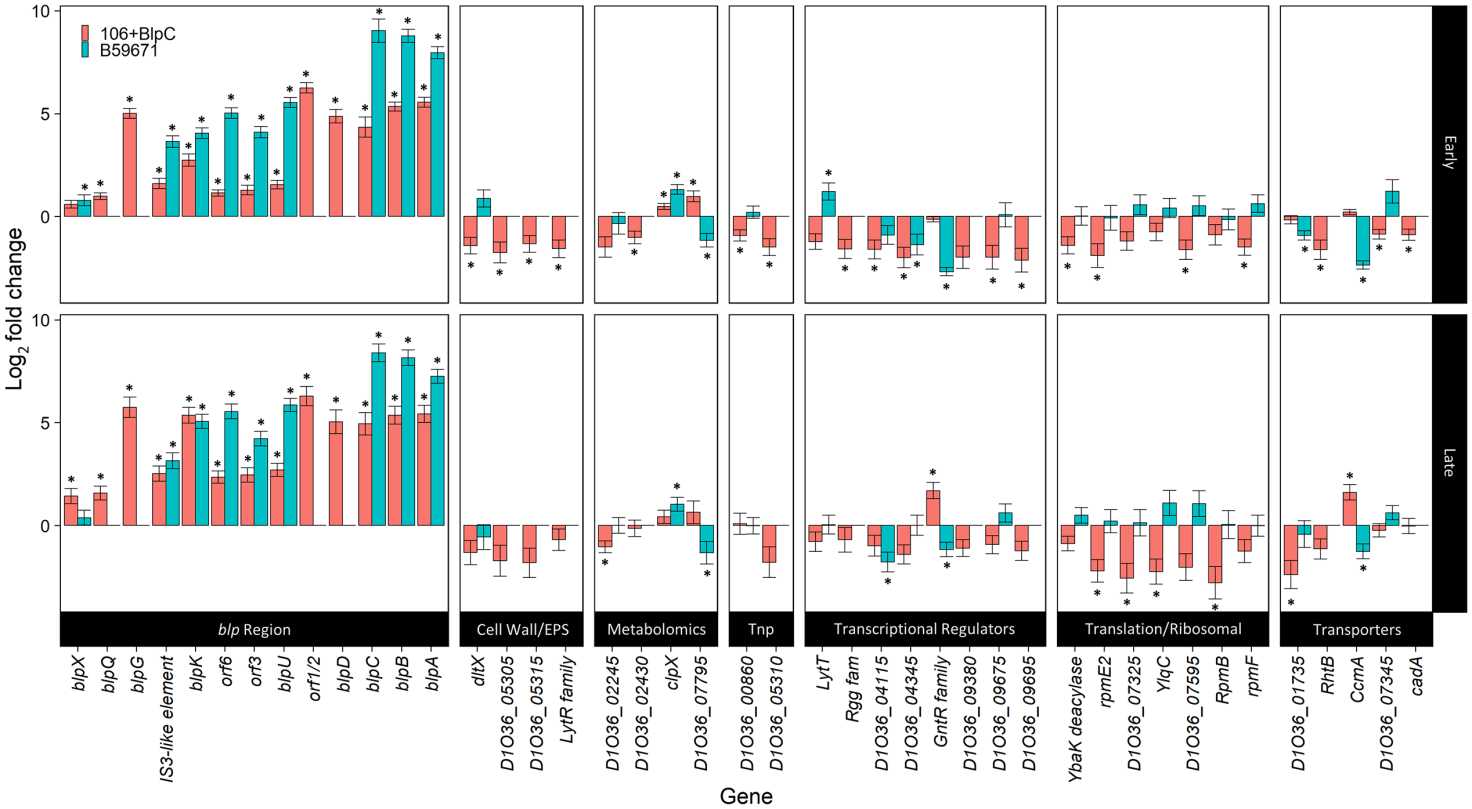
Differentially expressed genes during BlpC induction of ST106. DESeq2 analyses of ST106 induced with BlpC compared to ST106 not induced with BlpC (106B) and B59671 compared to ST106 not induced with BlpC (B59671) are shown. Means from three biological replicates are shown, and error bars represent standard errors. ^*^q less than 0.05.

RNA-Seq was performed on *S. thermophilus* ST106 cells collected in early and late exponential phases from BlpC-induced and non-induced cultures to identify all genes that are transcriptionally regulated by the *blp* quorum sensing signal peptide. As expected, the addition of exogenous BlpC significantly increased the transcription of the *blp*ABC and *blpD-orf*1/2 operons, *blp*K by approximately 10–35-fold in both early and late exponential phases, and *blp*U-*orf*3 to a significantly lesser extent (2.7–4.8-fold; Figure 2). BlpC was also shown to induce the transcription of *blpG*, *blpQ*, and *blpX* in both early and late exponential phases, with *blpG* transcription being comparable to what was observed for *blpA*, *D*, and *K*. The final gene that responded to BlpC induction encoded a transposase located upstream of *blpG*. Since the RNA-seq analyses took into account the DNA strand from which reads originated, it was concluded that read-through transcription from the upstream *blpK* was not responsible for the increased transcription of the transposon. Finally, transcription of the two-component sensor histidine kinase system encoded by *blpR* and *blp*H was not induced by BlpC.

There were 48 genes outside of the *blp* cluster that were identified as differentially expressed following BlpC induction when DESEeq2 was used for transcription analysis (Figure 2). Of this subset of differentially expressed genes, 19 encoded hypothetical proteins with unknown function (Supplementary Table 2), but D1O35_06360 and D1O35_07345 had predicted lipoprotein signal peptide domains. Of the remaining genes, transcription increased significantly for two genes in the early exponential phase, *clpX* and D1O36_07795, encoding a putative phosphoribosyl anthranilate isomerase involved in tryptophan biosynthesis (Hommel et al., 1995), and for D1O36_07100 and *ccmA*, which co-transcribe as part of a three-gene operon, in the late exponential phase. D1O36_07100 encodes a GntR-family/YtrA-subfamily transcriptional regulator, and *ccmA* encodes for one of the two ABC transporters within this operon. Transcription was downregulated for the remaining genes affected by BlpC induction in both early and late exponential phases (Figure 2) and included genes involved in the cell wall and extracellular polymeric substance synthesis; nucleobase (D1O36_01735) and heavy metal transport (*cadA*; D1O36_09675); protein translation; and six additional transcriptional regulators (*lytT* D1O36_02775; Rgg-family regulator D1O36_03425; D1O36_04115; D1O36_04345; D1O36_09675; and D1O36_09695).

Additional analysis of the RNA-seq data was performed using RT-qPCR and confirmed the upregulation of the operon consisting of genes encoding a GntR/YtrA-family transcriptional repressor and two membrane transporters.

### Differences in the transcription of bacteriocin-related genes between ST106 and B59671

Expression of genes from within the *blp* cluster continued to be higher in B59671 when compared to ST106 induced with exogenous BlpC during both early and late exponential phases (Figure 2, blue bars). Compared to ST106 (with or without exogenous BlpC), B59671 expresses *blpH* significantly higher (at least 2.5-fold) during both early and late exponential phases, and *blpR* significantly higher (over 4-fold) only during the early exponential phase.

Transcription of the genes outside of the *blp* gene cluster identified as being responsive to BlpC induction in ST106 was also assessed in B59671 by RNA-seq. Transcription patterns for genes associated with translation differed significantly between B59671 and BlpC-induced ST106, with transcription being higher in B59671. For *clpX*, transcription in B59671 was significantly higher than in ST106, and following the induction of ST106 with BlpC, the transcription of *clpX* increased but never reached the level observed in B59671. Additionally, the Rgg-family or XRE-family regulators that were downregulated in BlpC-induced ST106 were either not present or slightly upregulated (D1O36_09575) in B59671. Finally, a BLAST search revealed a *ytrA* homolog (CG712_RS03270) in B59671 with a 98.4% amino acid identity. In ST106, the YtrA-family regulator was upregulated in the late exponential phase only during BlpC induction and upregulated compared to B59671 in both early and late exponential phases regardless of BlpC induction.

Further analysis of the transcriptomic data for both ST106 and B59671 identified several clusters encoding ATP transport systems, histidine kinase/sensor regulator systems, and other products with potential bacteriocin-related functions that were actively transcribed in both strains (Table 1). A homolog to *blpA* was identified upstream of a gene encoding an ATP-binding protein (CG712_RS07685 and CG712_RS07690) only in B59671, with both genes being transcribed to a large extent. Another two sets of genes were identified as encoding for ABC transporters, sensor histidine kinases, and/or other proteins potentially related to bacteriocin production, processing, and transport in both strains: CG712_RS08380 (D1O36_02385), which is not differentially expressed between the two strains; and CG712_RS08745-CG712_RS08765 (D1O36_02770-D1O36_02790), with which genes encoding for ABC transporters and a bacteriocin adenylyltransferase are expressed by ST106 greater than 32-fold compared to B59671, and genes encoding for a sensor/histidine kinase are not differentially expressed between the two strains. Finally, B59671 was shown to possess a truncated gene cluster related to lantibiotic production, which contained genes potentially encoding for a lantibiotic dehydratase, a lanthionine synthetase, and an MFS transporter. Both strains also contained another putative bacteriocin gene (CG712_RS05160 and CG712_RS10045) but with a low raw read count, suggesting no significant expression in either strain.

**Table 1:** Expression of bacteriocin-related genes in *Streptococcus thermophilus* strains ST106 and B59671.

Gene accession number: B59671 (CG712_RS…)	Gene accession number in ST106 (D1036_…)	Function	Log_2_ fold-change	*q* value	RPKM in B59671
05160	09040	Class IIb bacteriocin, lactobin family	1.48	1.96E-3	46 ± 7
06650	^*^	Lantibiotic dehydratase	^*^	^*^	65 ± 6
06655	Unannotated	Lanthionine synthetase	2.23	3.44E-23	46 ± 3
10,400	09390	MFS transporter	−1.17	5.99E-6	997 ± 116
07685	^*^	Bacteriocin transporter ATP binding (79% identity/90% similar to BlpA)	^*^	^*^	387 ± 42
07690	^*^	ATP-binding protein	^*^	^*^	14 ± 1
08365	^*^	SH3 domain-containing protein	^*^	^*^	28 ± 4
08375	Unannotated	SH3 domain containing protein?	0.48	0.126	54 ± 5
08380	02385	BlpA (Kanehisa et al., 2023)	0.47	0.167	70 ± 24
08765	02790	Bacteriocin export ABC transporter permease	−5.19	5.12E-16	51 ± 17
08760	02785	Bacteriocin export ABC transporter ATP-binding	−5.37	1.17E-11	59 ± 21
08755	02780	Bacteriocin adenylyltransferase	−5.65	2.21E-12	194 ± 27
08750	02775	QS response regulator	0.01	0.993	157 ± 13
08745	02770	QS sensor kinase	0.02	0.971	3,161 ± 376
09525	^*^	Blp family class II bacteriocin	^*^	^*^	1,549 ± 172
09555	^*^	ThmS LytTR-type transcription regulator	^*^	^*^	1,484 ± 365
09560	^*^	ThmR (34% identity, 53% similar to BlpR)	^*^	^*^	661 ± 16
09565	^*^	ThmH (45% identity, 64% similar to BlpH)	^*^	^*^	39,472 ± 7,878
09570	^*^	ThmX (BlpC family pheromone)	^*^	^*^	570 ± 60
09575	^*^	ThmY (56% identity, 77% similar to BlpB)	^*^	^*^	320 ± 46
09580	^*^	Thermophilin transport ThmZ (79% identity, 90% similar to BlpA)	^*^	^*^	1,239 ± 225
09585	^*^	Thermophilin 13 (100% identity to ThmA of *S. thermophilus* SFi13)	^*^	^*^	28,726 ± 4,475
09590	^*^	Thermophilin 13 enhancer peptide (100% identity to ThmB of *S. thermophilus* SFi13)	^*^	^*^	166 ± 14
10045	04290	Annotated as “bacteriocin” (Benson et al., 2013)	−0.21	0.59	46 ± 7

Mean Log_2_ fold-changes represent late exponential phase transcription from B59671 compared to ST106, and all values are from DESeq2 analyses using read mappings to the B59671 chromosome. Homology in terms of identity and similarity is calculated using the amino acid sequence of the gene product from B59671. Means and standard errors between three biological replicates of B59671 in the late exponential phase are reported for the reads per kilobase of exon per million reads mapped (RPKM). ^*^Gene not present on the ST106 chromosome.

B59671 was also reported to have an intact locus that encodes for the previously characterized thermophilin 13 (Salini et al., 2023), and these genes were not found in ST106 (Table 1). B59671 expressed all genes within the *thm* cluster: *thmA* (CG712_RS09590) and *thmB* (CG712_RS09585) encoding thermophilin 13 and an enhancer peptide, along with *thmZ* (CG712_RS09580), *thmY* (CG712_RS09575), *thmX* (CG712_RS09570), *thmH* (CG712_RS09565), and *thmR* (CG712_RS09560), which function similarly to *blpA*, *blpB*, *blpC*, *blpH*, and *blpR,* respectively. In addition, a potential homolog to the BlpS regulator from *S. pneumoniae* (de Saizieu et al., 2000) was identified within the *thm* gene cluster (CG712_RS09555), which we designated as *thmS* (Proutiere et al., 2021).

### Effects of a *∆blpC* mutation on the transcription of bacteriocin-related genes in B59671

**Figure 3:**
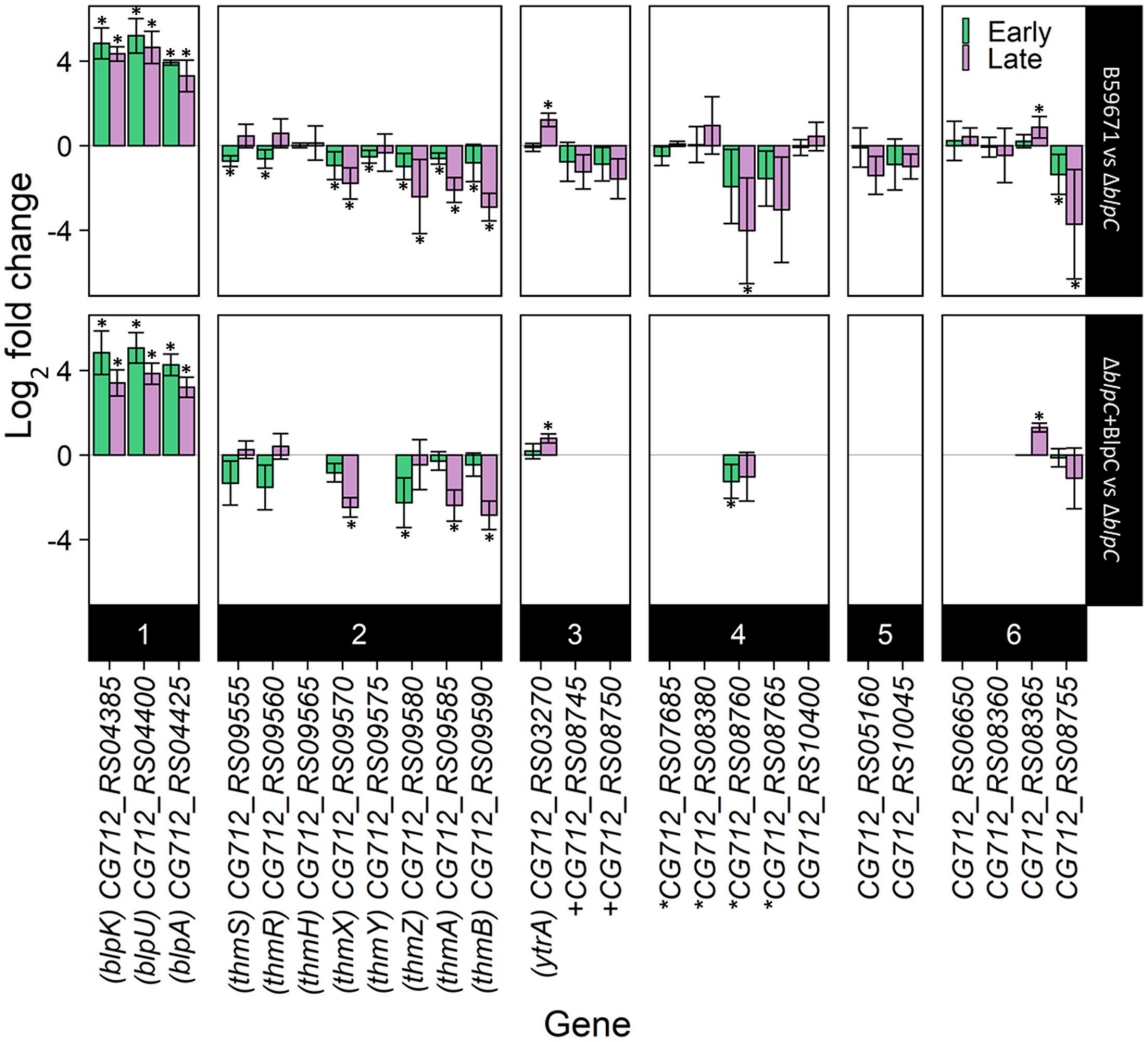
RT-qPCR results of bacteriocin-related genes in B59671 and B59671 *∆blpC*. B59671, B59671 *∆blpC* (*∆blpC*), and B59671 ∆*blpC* supplemented with BlpC (*∆blpC* + BlpC). Genes are categorized according to function as part of the *blp* cluster (1), part of the *thm* cluster (2), encoding a transcriptional regulator (3), encoding a transporter (4), encoding a putative bacteriocin-associated gene (5), or other (6). Means from three biological replicates are shown, and error bars represent 95% confidence interval bounds. Within the gene labels, ^*^gene encoding part of an ABC transporter + gene encoding part of a histidine kinase/response regulator. Within the chart area, ^*^q less than 0.05.

Since the RNA-seq data suggested BlpC-regulated transcription of genes outside the *blp* cluster in ST106, the potential for BlpC to regulate the expression of other bacteriocin loci in B59671 was investigated using a previously generated blpC knockout mutant (∆*blpC*; Somkuti and Renye, 2014). Transcription of several genes from within the thermophilin 13 cluster (*thmA*, *thmB*, *thmZ*, *thmY*, *thmX*, *thmR*, and *thmS*) and genes from another cluster (CG712_RS08755 and CG712_RS08760) was lower in the parent B59671 strain compared to the ∆*blpC* mutant (Figure 3). The gene CG712_RS08365 (*blpA* homolog) followed the opposite transcription pattern, with increased transcription in the wild-type background. Complementation of the ∆*blpC* mutant with exogenous BlpC restored the expression of *blpA*, *blpU*, *blpK*, *thmA*, *thmB*, *thmZ*, and *thmX* (ThmX acts as the QSIP for the *thm* cluster), and CG712_RS08365 (*p* < 0.05) to what was observed in the parent strain. Supplying BlpC exogenously did not significantly restore the transcription of CG712_RS08755 or inhibit the transcription of *thmR* or *thmS*. Transcription of the Ytr*A-*family regulator was identified as being regulated by BlpC in ST106. Thus, its transcription was assessed in the B59671 mutant and shown to be more increased in the parent B59671 strain compared to the ∆*blpC* mutant (Figure 3).

**Figure 4:**
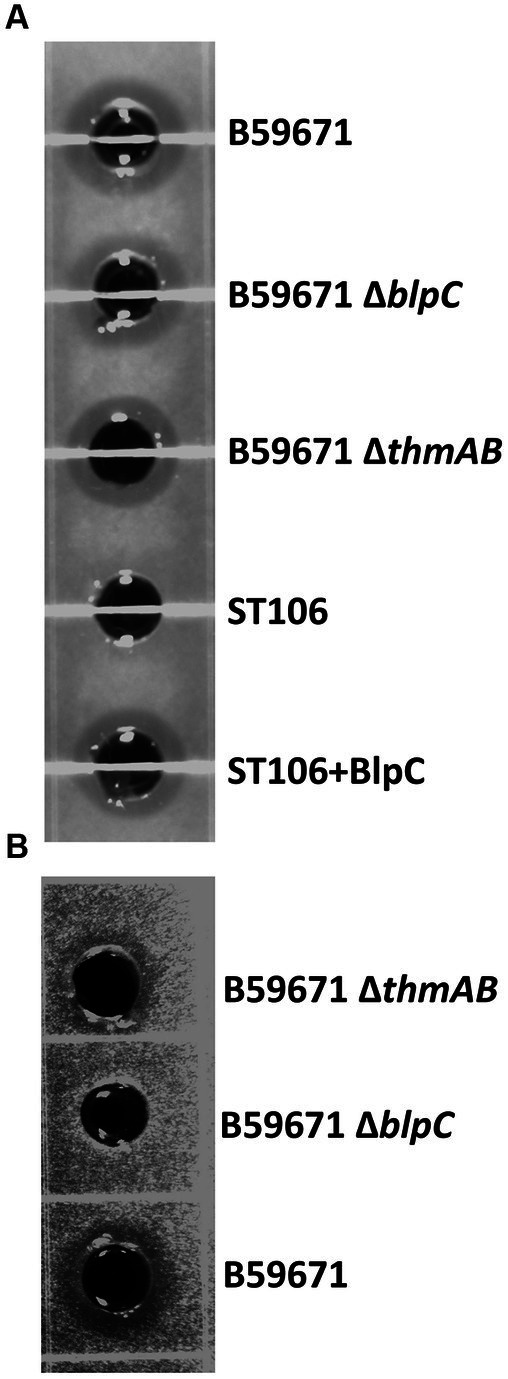
Representative supernatant zones of inhibition from cell-free supernatants from the listed strains against target **(A)** *Streptococcus thermophilus* ST113 and **(B)** *Cutibacterium acnes* ATCC6919. +BlpC induced with BlpC.

With thermophilin 13 production appearing to increase in the ∆*blpC* mutant, the mutant was screened for antimicrobial activity against a variety of targets known to be susceptible to thermophilin 110. Antimicrobial activity against *Pediococcus acidilactici* F was not previously observed with the ∆*blpC* mutant (Renye and Somkuti, 2013), and in this study, the mutant could not inhibit the growth of *C. acnes* (Figure 4). However, the ∆*blpC* mutant was still active against *S. thermophilus* ST113 (Figure 4). To test if these activities were dependent on the production of thermophilin 13, a ∆*thmAB* mutant was generated and screened for antimicrobial activity. This mutant, however, maintained activity against ST113 (Figure 4).

### Effects of bacteriocin production on growth in B59671 and ST106

**Figure 5:**
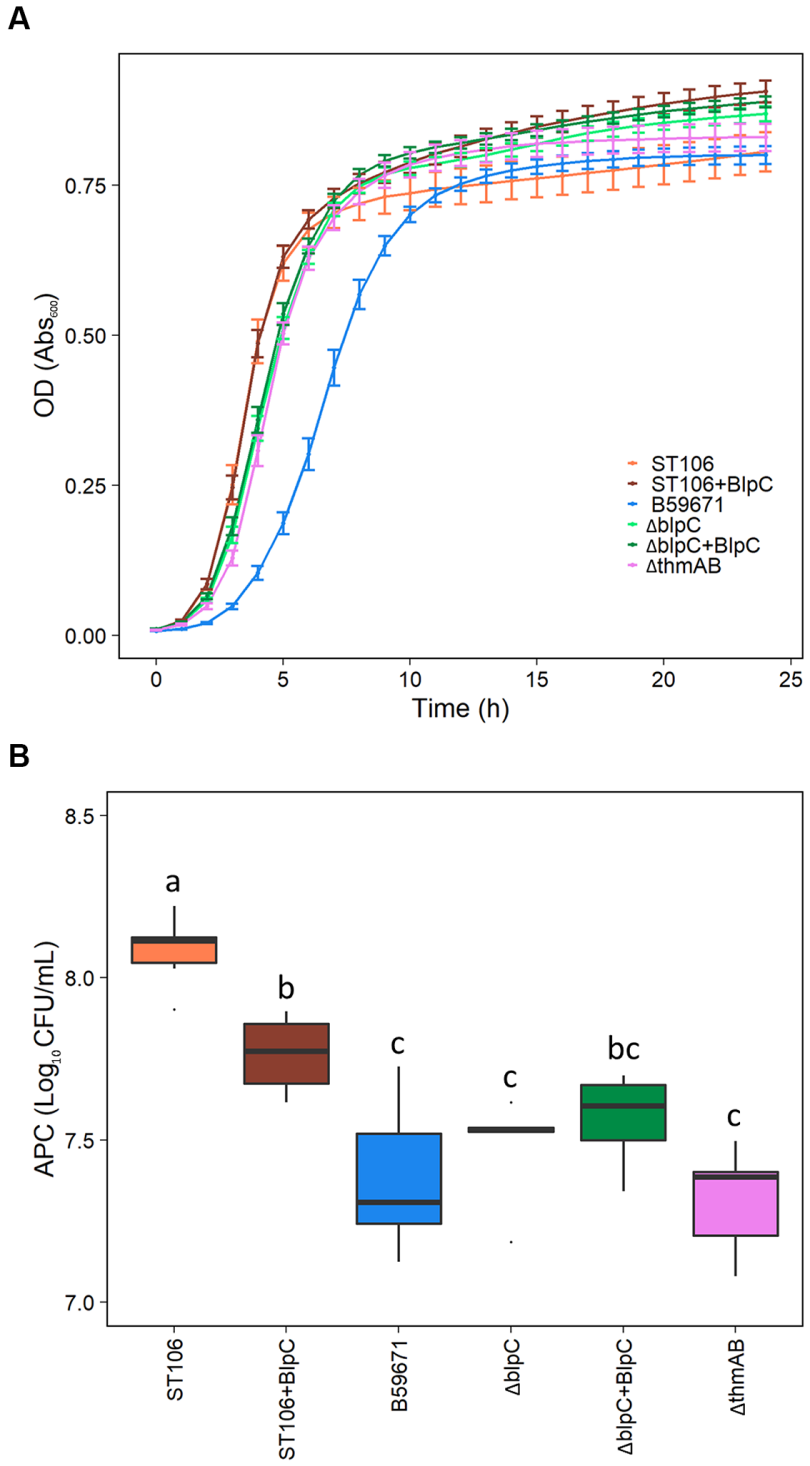
**(A)** Growth curves, and **(B)** APC after 24 h growth. Averages and standard errors between six biological replicates are shown for ST106, ST106 induced with BlpC (ST106 + BlpC), B59671 (ST110), B59671 ∆*blpC* (∆blpC), B59671 ∆*blpC* induced with BlpC (∆blpC+BlpC), and B59671 ∆*thmAB* (∆thmAB).

**Figure 6:**
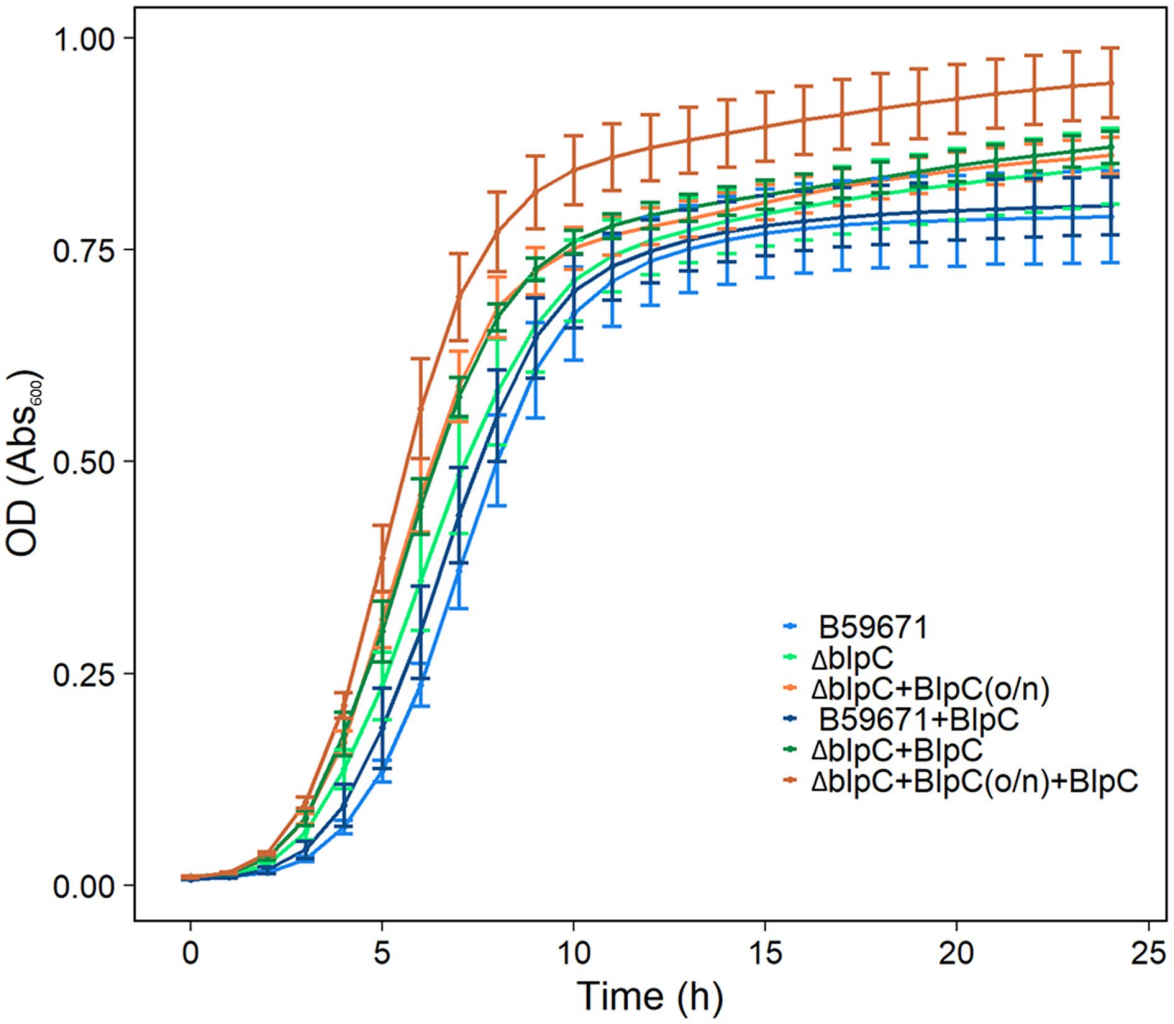
Growth curves of cultures induced/not induced with BlpC in the inoculum and tested cultures. Averages and standard errors between six biological replicates are shown for B59671, B59671 ∆*blpC* (∆blpC), B59671 ∆blpC with exogenous BlpC in the inoculum but not tested culture [∆blpC+BlpC(o/n)], B59671 with exogenous BlpC (B59671 + BlpC), B59671 ∆*blpC* with exogenous BlpC in the tested culture but not in the inoculum (∆blpC+BlpC), and B59671 ∆*blpC* with exogenous BlpC in the inoculum and in the tested culture (∆blpC+BlpC(o/n) + BlpC).

B59671 and its ∆*blpC* and ∆*thmAB* mutants were assayed to determine if bacteriocin production caused any impacts on growth. As shown in Figure 5A, B59671 experiences a growth defect in terms of a longer lag phase than ST106. This growth defect is likely due to its bacteriocin production, since both B59671 ∆*blpC* and B59671 ∆*thmAB* mutants grew faster than wild-type B59671 and were more similar to ST106. CFU counts (Figure 5B) indicated that B59671, along with ST106 supplemented with BlpC, had lower stationary-phase CFU counts than ST106. Biomass in terms of OD_600_ from ST106 induced with BlpC followed a different trend, with BlpC-induced cultures reaching higher stationary-phase OD_600_ than non-BlpC-induced cultures (*p* = 0.014 at 24 h, Figure 5A). Supplementation of B59671 ∆*blpC* with BlpC caused insignificant changes to growth (*p* = 0.981 at 24 h, Figure 5A), and adaptation of the strain by inducing the overnight culture with BlpC also caused insignificant changes to growth (*p* = 0.078 at 24 h, Figure 6). Unexpectedly, B59671 ∆*blpC*, with or without exogenous BlpC, and B59671 ∆*thmAB* were not significantly different from wild-type B59671 in terms of viability (*p* = 0.078 at 24 h, Figure 5B).

Another interesting phenomenon occurred under conditions in which *blp* bacteriocins are naturally produced without the aid of exogenous BlpC. All other cultures continue a small linear increase in OD_600_ in the stationary phase for at least 24 h, while B59671 and B59671 ∆*thmAB* completely stop increasing OD_600_ within 19 h of growth.

## Discussion

In this study, BlpC supplementation with ST106 increased the transcription of several genes within the *blp* gene cluster, similar to what was reported previously (Somkuti and Renye, 2014). The *blpABC* and *blpD*-ORF1/2 operons and *blpK* were significantly more induced than the *blpU*-ORF3 operon. This is consistent with results from strain LMD-9, in which BlpC induces transcription from *blpD* and *blpE* much more than from *blpU* (Fontaine et al., 2007), and the purported BlpR-binding sites in the promoters for *blpD*, *blpU*, and *blpK* in ST106 are identical to those for *blpD*, *blpU*, and *blpE*, respectively, in LMD-9. BlpG may be required for the formation of disulfide bonds between antimicrobial peptides (BlpD, BlpU, BlpE, and BlpF) in strain LMD-9 (Fontaine and Hols, 2008). In ST106, *blpG* transcription was comparable to that of both *blpD* and *blpK*, suggesting a similar role for BlpG in this strain. Not reported previously was the induction of transposase within the *blp* cluster. The same transposase was identified in the same location within the *blp* loci of *S. thermophilus* strains B59671, ST109, and LMG18311 (Fontaine et al., 2007; Renye and Somkuti, 2017; Renye et al., 2019b), but its function remains unknown. Finally, our results also confirm previous reporting of constitutive expression of the *blpRH* operon (Fontaine et al., 2007; Somkuti and Renye, 2014).

Our findings that BlpC affects the expression of genes outside of the *blp* cluster in *S. thermophilus* ST106 and B59671 contrast with a study of the *blp* system in *S. pneumoniae*, in which microarray data revealed that BlpC only induces genes within the *blp* cluster (de Saizieu et al., 2000). With the limitations of microarray analyses compared to the RNA-seq analyses employed here, it is still possible that BlpC induces genes outside of the *blp* cluster as well in *S. pneumoniae*. Of the ST106 genes identified as being regulated by BlpC in this study (Supplementary Table 2), *clpX* is one that has been reported to affect bacteriocin production. In *Streptococcus mutans,* it was shown that the deletion of *clpX*, an ATPase subunit of the Clp protease complex, resulted in a loss of antimicrobial activity (Kajfasz et al., 2011), but the mechanism by which ClpX regulates bacteriocin production remains unknown. Additionally, three of the transcriptional regulators identified were putative xenobiotic response element (XRE)-family transcription factors (D1O36_04345, D1O36_09575, and D1O36_09695), and one was a Rgg-family regulator, and examples of each serving as repressors in other streptococcal species have been reported (Capodagli et al., 2020; Liu et al., 2020). BlpC induction lowered the transcription of these regulatory factors in the early exponential phase, suggesting that they could serve to repress bacteriocin production in ST106. Of particular interest would be the XRE-family regulator D1036_04345, as it was transcribed lower in B59671 when compared to uninduced ST106. Reduced transcription of this gene could be required for bacteriocin production from within the *blp* locus to occur in *S. thermophilus*. However, to conclude if any of these regulators truly affect bacteriocin production, the generation of knockout mutants and subsequent phenotype analysis are required.

The operon encoding a putative YtrA-family transcriptional regulator in *S. thermophilus* ST106 that is upregulated during BlpC induction organizationally resembles a three-gene operon identified in *Sulfolobus solfataricus* (Lemmens et al., 2019). In other bacterial species, homologs of *ytrA* are within operons consisting of two or six genes in *Sulfolobus* species and *Bacillus subtilis*, respectively (Salzberg et al., 2011). YtrA was reported to regulate cell envelope stress responses in response to cell wall antibiotics in *B. subtilis* (Salzberg et al., 2011) and repress the transcription of its own operon and a specific subset of genes encoding membrane proteins in *S. solfataricus* (Lemmens et al., 2019). Bacteriocins encoded within the *S. thermophilus blp* gene cluster are believed to exert their antimicrobial activity by forming pores within the target cell membrane (Gilbreth and Somkuti, 2005; Renye et al., 2023). Therefore, it is reasonable to hypothesize that the YtrA-family regulator in *S. thermophilus* is part of a stress response associated with the production of bacteriocins.

The results from this study identified significant differences between ST106 and B59671 that could explain why ST106 induced with BlpC still transcribes *blp* genes less than B59671 does (Figure 2). The differences in the expression of the histidine kinase (*blpH*) and response regulator (*blpR*) could allow B59671 to be more sensitive to BlpC, resulting in the increased transcription of BlpC-regulated genes. The higher expression of *clpX* may contribute to the natural production of thermophilin 110 by strain B59671, and the low-level transcription of potential repressors, including D1O36_04116 and 04345, and the YtrA-family regulator may be needed to begin a sequence of events required for bacteriocin expression.

This is the first study to show that BlpC regulates the expression of bacteriocins outside the *blp* gene cluster in *S. thermophilus* (Figure 1B). Our data clearly show cross-talk between the *blp* and *thm* gene clusters, in that BlpC downregulates transcription of genes from the *thm* bacteriocin cluster. We also show that BlpC upregulates (CG712_RS08365) and downregulates (CG712_RS08760) genes from within other clusters. On the reverse, B59671 may depend on the alternative homologs of *blpA*, *blpB*, *blpR*, and *blpH* (i.e., *thmZ*, *thmY*, *thmR*, and *thmH*), as well as an increased expression from *blpR* and *blpH*, to amplify the quorum sensing signal to induce *blp* bacteriocin expression. Further research is needed to determine if the BlpA and BlpB homologs encoded by genes outside the *blp* cluster interact with BlpC, BlpD, BlpU, and BlpK, and if the BlpR and BlpH homologs encoded by genes outside the *blp* cluster interact with BlpC, BlpR, and BlpH.

In the related *S. pneumoniae blp* system, four distinct *blpH* alleles and *blpC* types have been identified (Pinchas et al., 2015). Our study represents a case where more than one *blpH-* and *blpC*-type system is actively expressed by the same host. As for other *blp-*related genes, the finding of a *blpS* homolog (*thmS*) only in B59671 was unexpected. While BlpS has an unknown role in *S. pneumoniae* (de Saizieu et al., 2000), BlpS purportedly acts to repress the transcription of *blp* genes in *Streptococcus gallolyticus* (Proutiere et al., 2021). It is possible that the BlpS homolog only represses genes from the *thm* cluster, since that is where it is located in the chromosome.

Our results confirm that a *blpC* knockout mutant of *S. thermophilus* B59671 (∆*blpC*) lost its broad-spectrum antimicrobial activity against *P. acidilactici* F (Renye and Somkuti, 2013). RT-qPCR data from this study confirmed that the antimicrobial activity in the B59671 parent strain was due to the increased expression of genes within the *blp* locus, specifically *blpA*, *blpU*, and *blpK* compared to the ∆*blpC* mutant (Figure 4). The antimicrobial activity could be restored by inducing the expression of the *blp* gene cluster with exogenous BlpC (Somkuti and Renye, 2014). However, in this study, when *S. thermophilus* ST113 was used as a target, inhibition zones were observed, suggesting that a second active bacteriocin was being produced. An *in silico* study identified the presence of the *thm* gene cluster encoding thermophilin 13 in B59671 (Salini et al., 2023), but no studies were performed to confirm the expression of this bacteriocin. This is the first study to show that the genes encoding thermophilin 13 are expressed in B59671. Unfortunately, the *ΔthmAB* mutant generated did not show a clear phenotype. The mutant still inhibited the growth of *S. thermophilus* ST113, *P. acidilactici*, and *C. acnes*. These results suggest that thermophilin 110 alone is responsible for the unique broad-spectrum activities previously reported for strain B59671, which include activities against *Enterococcus faecalis*, *Enterobacter faecium*, *Streptococcus mutans*, *Streptococcus pyogenes*, *Streptococcus salivarius*, *Lactobacillus acidophilus*, *Lactobacillus helveticus,* and *Cutibacterium acnes* (Renye and Somkuti, 2013, 2017; Renye and Steinberg, 2021; Renye et al., 2023). The optimal intraspecies activity of this strain may be due to the production of both thermophilins since our results suggest they are both expressed. The redundancy of their activities would also prevent a loss of intraspecies activity if a random mutation were to occur within one of the gene clusters, allowing them to maintain a competitive advantage in the presence of other *S. thermophilus* strains. To confirm that intraspecies activity is dependent on both systems, it would require the generation of a double mutant with both systems inactivated.

It appears that the production of two active bacteriocins by B59671 comes at a cost, as the parent strain had a longer lag phase and lower final biomass when compared to either the *ΔblpC* or *ΔthmAB* strains. Indeed, fitness costs have been modeled (Lehtinen et al., 2022) and demonstrated for bacteriocin production in *Lactobacillus plantarum* (Maldonado-Barragan and West, 2020), and even bacteriocin immunity in *Listeria monocytogenes* (Dykes and Hastings, 1998). In ST106, BlpC induction results in the production of an active bacteriocin but does not impact exponential growth, potentially due to both the histidine kinase (BlpH) and response regulator (BlpR) being expressed ~2-fold lower in the early exponential phase. In the late exponential phase, when these components are expressed at a higher level, a phenotype is observed with the uninduced culture entering the stationary phase at a reduced biomass. The growth-impaired phenotype observed for the parent B59671 culture may be due to both the *blp* and *thm* bacteriocin gene clusters being highly expressed in the early exponential phase, which requires the bacterium to continuously expend energy producing other components within these gene clusters required for processing and secretion of the signal peptides, a purported immunity protein (orf 3) (Fontaine et al., 2007), and bacteriocins (*blpA* and *blpB*). Additionally, our results suggested that cross-talk occurs between the *blp* and *thm* systems, but the effect of these systems on the overall transcriptome of B59671 is not known. The results from this study showed that the transcription of several genes outside the *blp* gene cluster was affected by BlpC induction within ST106. Some of these same genes appeared to be regulated by BlpC in B59671, specifically the YtrA-like transcriptional regulator (Figure 3). The function of this regulator is unknown in *S. thermophilus*, and more studies are required to demonstrate if it affects bacterial growth. It is also possible that the signaling peptides BlpC or ThmX regulate the transcription of other genes within B59671 that contribute to the impaired exponential growth observed. The construction of additional mutant strains is required to accurately assess the role of each signaling molecule with respect to cell growth and metabolism.

Bacteriocin production has long been thought to provide an advantage when a bacterium is attempting to establish itself within a specific niche by eliminating bacteria that are competing for the same nutrients. The broad-spectrum activities of bacteriocins produced by lactic acid bacteria have also been reported to drive intra-guild predation, allowing them to obtain nutrients from the lysis of other bacterial species to ensure their survival (Leisner and Haaber, 2012). Additionally, bacteriocins may aid in the survival of a sub-population within the same species or strain. While such an effect is reminiscent of persistence, which temporarily allows sub-populations of genetically identical bacteria to survive exposure to antibiotics at bactericidal concentrations (Balaban et al., 2019), further experiments would be needed to determine if persistence is involved. In any case, the intracellular accumulation of the bacteriocin CipB in *Streptococcus mutans*, which occurs in response to the detection of competence-stimulating peptides, leads to self-autolysis (Perry et al., 2009). This altruistic cell death was proposed to ensure the survival of a subset of the population and potentially increase the genetic diversity of the surviving population via the uptake of released extracellular DNA (eDNA) (Perry et al., 2009). In *S. pneumoniae,* bacteriocins encoded within the *blp* gene cluster decrease population diversity through intra-strain predation (Aggarwal et al., 2023). This selfish killing of kin cells resulted in a sub-population of bacteriocin-producers as the predominant colonizers within a murine infection model (Aggarwal et al., 2023). In this study, the expected decrease in biomass following BlpC induction in ST106 and the *ΔblpC* mutant of B59671 could have been obscured by death in a sub-population of bacteriocin non-producers, causing the release of nutrients and growth of the bacteriocin-producing sub-population. Our results suggest that the expression of chromosomal *blpC* causes more of a detriment to cell growth than supplementation with extracellular BlpC, but more studies are needed to determine the cause of this and if intraspecies predation truly contributes to the growth differences observed.

Altogether, the growth phenotypes observed here indicate that there is selective pressure to not produce bacteriocins and/or BlpC. Competition with bacteria susceptible to the bacteriocins causes the opposing selective pressure to produce bacteriocins. ST106 and other strains that lack the capacity to produce bacteriocins without externally supplied BlpC may have gained a dependence on other strains to produce BlpC or evolved in environments where growth rate was more important than bacteriocin production in terms of cell growth and survival. A similar system was found in *S. pneumoniae*, whereby some “producer” strains produce bacteriocin and “cheater” strains carry a conserved frameshift mutation that renders a non-functional *blpA*, allowing them to sense the QSIP and produce immunity proteins but not to secrete bacteriocins and QSIP (Son et al., 2011). ST106 compromises by retaining the ability to produce and secrete its bacteriocins in the presence of QSIP.

## Data availability statement

The RNA-seq data presented in the study are deposited in the NCBI Sequence Read Archive (SRA), accession number PRJNA1021576.

## Author contributions

MM: Conceptualization, Formal analysis, Investigation, Methodology, Writing – original draft. GG: Data curation, Formal analysis, Visualization, Writing – review & editing. AO: Formal analysis, Investigation, Writing – review & editing. AM: Visualization, Writing – review & editing. JR: Conceptualization, Supervision, Writing – review & editing.
