# Supplementary material for: The quorum sensing peptide BlpC regulates the transcription of genes outside its associated gene cluster and impacts the growth of Streptococcus thermophilus
Source: Front Microbiol. 2024 Jan 8;14:1304136. doi: 10.3389/fmicb.2023.1304136 (PMC10826417; doi:10.3389/fmicb.2023.1304136)
Supplement: Supplementary file 1 [file Table_1.DOCX]

***Supplementary Material***

**Supplementary Table 1.** Primers used in this study.

| **Primers used for qPCR** | | | | | |
| --- | --- | --- | --- | --- | --- |
| Gene name | Gene ID in ST106 | Gene ID in B59671 | Forward primer | Reverse primer | Notes |
| *rlmD* | D1O36_01975 | CG712_RS07980 | 5’-TGGCCAAGAGACAGAAATTATC | 5’-AGGAGAAAGTGCGAAGTTATAG | Reference |
| *blpA* | D1O36_08315 | CG712_RS04425 | 5’-TCCGCTCAGCGATACTAAG | 5’-GCAACTAGCGGATTGGATG |  |
| *blpB* | D1O36_08310 | CG712_RS04420 | 5’-ACCTTGGTTGGCTCTATACTTC | 5’-GTCGCTACCACAATATGTCTAC |  |
| *blpC* | D1O36_08305 | CG712_RS04415 | 5’-ACCACGCTCTTGAACAAGTC | 5’-TTTGTTGGAAGCGTTCTTTGC |  |
| *blpH* | D1O36_08300 | CG712_RS10255 | 5’-GTGCTTCAAGAATCCGATAAAC | 5’-ATCTCCATCCCAATCAAGTG |  |
| *blpR* | D1O36_08295 | CG712_RS04405 | 5’-TGGCGTTACAGCCTATAAATAC | 5’-TAGTTGCGTTAATAGGGAAGAC |  |
| *blpD* | D1O36_08290 |  | 5’-TATAAGGCTGCACCTACTAACACC | 5’-CTCGAAACACTTGCTAGTGTTGAG |  |
| *blpU* | D1O36_08280 | CG712_RS04400 | 5’-GGTAATGGTTTACGACTAGGTATC | 5’-ATGTTGCTCCATAACCAACAC |  |
| *blpK* | D1O36_08265 | CG712_RS04385 | 5’-AGCAGGTTGCTCCATAGG | 5’-ACCAGTTGTAGGCTCAGTTC |  |
|  | D1O36_07100 | CG712_RS03270 | 5’-AGGAAGCCGGTGTCAATC | 5’-GACGTACGGGCAGAATAAAC |  |
|  | D1O36_07105 | CG712_RS03275 | 5’-TGCCCACCAATTAATCTTGTC | 5’-AGGTTGAAAGCGGCTATAATG |  |
|  | D1O36_07110 | CG712_RS03280 | 5’-CGTTGTACTCGCTTCAATTGG | 5’-TTTGACCGAAGTAGCTGGTAG |  |
| *pbuG* | D1O36_01735 |  | 5’-CAGAAGTGACTGCCGGATTG | 5’-GTGCAGGCATTCCTGTTTGG |  |
|  | D1O36_01960 |  | 5’-TCACTATCAGCCTGTAACTTTG | 5’-ACGGTAACGTTTCTCAGATCTC |  |
|  | D1O36_03620 |  | 5’-TCAGTAATGCGACTGGAAG | 5’-CTTTAACTATGCTGTCATAAATTGG |  |
|  | D1O36_03665 |  | 5’-AATGACTGGTCCTGTCTTATCC | 5’-GATAGCTTATCCTTCAACGGTTTAG |  |
|  | D1O36_03680 |  | 5’-CCCAAGGGACAATATAGTGAAC | 5’-CCTCATCACTGGAGTCATCTATC |  |
| *ridA* | D1O36_04225 |  | 5’-AGCGATTGGACCTTATGTTC | 5’-ACCTGTTTCTGGTGACAATG |  |
|  | D1O36_04410 |  | 5’-TTTGGGAGCCTTGCTTCAC | 5’-GGAAGGAGATACTTGACCAGAATAATG |  |
|  | D1O36_04625 |  | 5’-TATTTCCCGCTTTGAACGTG | 5’- GAAAGTCGTTTGCTGCATAC |  |
| *rpsU* | D1O36_07325 |  | 5’-AGCTGCCTCTGATTTACGTTTAC | 5’-CGTTTCAAACGTTCTGTGACTAAAG |  |
|  | D1O36_08610 |  | 5’-AATCTTTGGTTTCTCGGGAACAC | 5’-CATAGCAAGGCCACCAATAAATG |  |
|  | D1O36_09355 |  | 5’-AATACAGGTGCAGCATGGAGTG | 5’-AGTTACCTAACGTTCCAGCAATC |  |
|  | D1O36_09380 |  | 5’-GTAGTTGCTACTGTTGAATTTAC | 5’-AAAGCACTATCTGAAGAAACAAG |  |
|  | D1O36_09695 |  | 5’-TCGTCTCCATCTTCTGTGTATAG | 5’-CTAGAAACTGATGGACAGTACTTAG |  |
| *rpmB* | D1O36_09730 |  | 5’-ACGTGTCGTTAAGCCTAACC | 5’- TTTAAGTGCACGAGCTGAAG |  |
|  |  | CG712_RS05160 | 5’-CTACTCAAGAACTCGAAATCGTATC | 5’-ATTTACCCGCAGCATAGCTTAG |  |
|  |  | CG712_RS06650 | 5’-GAATTGCACATTGGATAGACTTC | 5’-TAAAGTTATCTCCACCACATCTC | Lantibiotic dehydratase |
|  |  | CG712_RS07685 | 5’-TGGGTAAGAAATCCTGACTAATAATTGTG | 5’-GTTCTTTATTTCGGTAGTTGGATTATTGG | *blpA* homolog |
|  |  | CG712_RS08360 | 5’-TTGGAGCTACATCAGTCTTGATTG | 5’-TCCTTGCTGGAACTTAAGCTATTG |  |
|  |  | CG712_RS08365 | 5’-TCGTAGGAGTGTCTGTTCCAATC | 5’-GGTCACTTAGACCGACATTAACC | SH3 upstream of *blpA* homolog |
|  |  | CG712_RS08380 | 5’-TTTGTGGTAGCCGTTAACCG | 5’-GCCTGCAAGACTACATTTCC |  |
|  |  | CG712_RS08745 | 5’-GCTCCTGCTAACAAACTTAGAC | 5’-GGAACTCGTGTTCCTATTCTC |  |
|  |  | CG712_RS08750 | 5’-CTACGTCAATTCCATTGATTCC | 5’-AGAGATGCTAGCCAATTAGAAC |  |
|  |  | CG712_RS08755 | 5’-GGACGTGTTTATTCTGTAGTACC | 5’-CCCGAAATTGTTCCAAGAATAG | Bacteriocin adenylyltransferase |
|  |  | CG712_RS08760 | 5’-CACAGCTCTTATAGGACATAATGG | 5’-GAAATATCTCCGTAGTGAACATCTC |  |
|  |  | CG712_RS08765 | 5’-TTGGGTATCCGTTGGTAATTG | 5’-ACTTGGCATTGGCTATAACAG |  |
|  |  | CG712_RS09555 | 5’-TATTAGATGCCATCGGTCTAGTC | 5’-CTGATGCAGTATGTCCTGATAAC |  |
|  |  | CG712_RS09560 | 5’-CCGAGTTTATGCCCTTATCTTTCAG | 5’-GGCGCTATTGTCCTTATTGTG |  |
|  |  | CG712_RS09565 | 5’-GCTTTATGCCTTTGGTCATAGTTC | 5’-TGCACTAGTCGTGTTAAGTGATAC |  |
|  |  | CG712_RS09570 | 5’-CTATGGCTTGCTTTGACTTAGG | 5’-ATGACCAAGCATCGAACTTCTC | *blpC* homolog |
|  |  | CG712_RS09575 | 5’-CCTGTGACTGGGTGATAAAGTTG | 5’-CCAGCTAGCCACTCTAAATAGAC | *blpB* homolog |
|  |  | CG712_RS09580 | 5’-AATGAGCTGAGCTCCCTGTTTAAG | 5’-ATTGATAGCGAGTTCGTAGATTACC |  |
|  |  | CG712_RS09585 | 5’-AAAGACATGGGAGGATATGC | 5’-ACCAATCATTCCACCCATAC |  |
|  |  | CG712_RS09590 | 5’-TGAACTTGACTTAGCAAATGTAAC | 5’-CGATAATAGCTCCACCTATACAG |  |
|  |  | CG712_RS10045 | 5’-GAACAAGCAACAGTCGTTTC | 5’-GCAAGTGTTAGGGTTTCTCTATC |  |
|  |  | CG712_RS10400 | 5’-AAACGTTATGGCTACGGGTACTG | 5’-TGTCCCAATGTATCTGCAACAAG |  |
| **Primers used for generating the ∆*thmAB* mutant and verifying** | | | | | |
|  |  |  | 5’-TAGCACATCAAGGCCGCTAG | 5’-CTGGCCGTCGTTTTACAACGTC TCAAGAAGGGAAAGCGACCG | Upstream of *thmAB* |
| *kanR* |  |  | 5’-CGGTCGCTTTCCCTTCTTGA GACGTTGTAAAACGACGGCC | 5’-GCTCCCAACAAGGCATCCTA CCTTTACAGAATTACTCTATG | *kanR* of pKS1 |
|  |  |  | 5’-CATAGAGTAATTCTGTAAAGG TAGGATGCCTTGTTGGGAGC | 5’-AGCATCATAGCCAGCCTGTG | Downstream of *thmAB* |
|  |  |  |  |  |  |
|  |  |  |  |  |  |
|  |  |  |  |  |  |

**Supplementary Table 2.** Genes in ST106 that were differentially expressed during BlpC induction according to DESeq2. Log_2_ fold changes and q values are reported for ST106 induced with BlpC compared to ST106 not induced with BlpC. Signal peptide probabilities using SignalP 6.0 (Teufel et al., 2022) are shown.

|  |  | DESeq2 results (early) | | DESeq2 results (late) | | SignalP 6.0 peptide probability | | | | | |
| --- | --- | --- | --- | --- | --- | --- | --- | --- | --- | --- | --- |
| Gene ID in ST106 | Gene product function | Log_2_ fold change | q value | Log_2_ fold change | q value | Other | Signal Peptide | Lipoprotein signal peptide | TAT Signal peptide | TAT lipoprotein SP | Pillin like |
| D1O36_00045 | recombinase | -1.60 | 0.027 | -0.36 | 0.848 | 1 | 0 | 0 | 0 | 0 | 0 |
| D1O36_00185 | rod shape determining protein MreC | -2.08 | 0.004 | -2.34 | 0.028 | 1 | 0 | 0 | 0 | 0 | 0 |
| D1O36_01735 | NCS2 family permease | -0.17 | 0.594 | -2.39 | 0.039 | 1 | 0 | 0 | 0 | 0 | 0 |
| D1O36_01820 | levansucrase | -0.72 | 0.039 | 0.10 | 0.943 | 1 | 0 | 0 | 0 | 0 | 0 |
| D1O36_01960 | DUF1803 domain containing protein | -2.11 | 0.016 | -1.72 | 0.069 | 1 | 0.0001 | 0 | 0 | 0 | 0 |
| D1O36_02245 | peptide deformylase | -1.48 | 0.065 | -1.04 | 0.031 | 0.9995 | 0.0005 | 0 | 0 | 0 | 0 |
| D1O36_02430 | alpha-L-glutamate ligase | -1.02 | 0.033 | -0.14 | 0.909 | 1.0001 | 0 | 0 | 0 | 0 | 0 |
| D1O36_02775 | DNA-binding response regulator | -1.22 | 0.043 | -0.79 | 0.712 | 1.0001 | 0 | 0 | 0 | 0 | 0 |
| D1O36_02980 | clpX | 0.49 | 0.039 | 0.42 | 0.741 | 1.0001 | 0 | 0 | 0 | 0 | 0 |
| D1O36_03080 | DUF1827 family protein | -1.68 | 0.028 | -0.56 | 0.741 | 1.0001 | 0 | 0 | 0 | 0 | 0 |
| D1O36_03290 | truncated hypothetical protein | -1.11 | 0.205 | -1.09 | 0.031 | 1 | 0.0001 | 0 | 0 | 0 | 0 |
| D1O36_03425 | Rgg/GadR/MutR family transcriptional regulator | -1.58 | 0.038 | -0.70 | 0.741 | 1 | 0 | 0 | 0 | 0 | 0 |
| D1O36_03615 | LysE family translocator | -1.62 | 0.033 | -1.14 | 0.365 | 1 | 0 | 0 | 0 | 0 | 0 |
| D1O36_03620 | hypothetical protein | -1.86 | 0.012 | -0.73 | 0.741 | 1.0001 | 0 | 0 | 0 | 0 | 0 |
| D1O36_03665 | aminoacyl-tRNA deacylase | -1.40 | 0.039 | -0.88 | 0.279 | 1 | 0 | 0 | 0 | 0 | 0 |
| D1O36_03905 | Type B 50S rbiosomal protein L31 | -1.90 | 0.043 | -2.21 | 0.006 | 1.0001 | 0 | 0 | 0 | 0 | 0 |
| D1O36_03985 | dltX | -1.42 | 0.025 | -1.32 | 0.408 | 1 | 0 | 0 | 0 | 0 | 0 |
| D1O36_04050 | hypothetical protein | -1.47 | 0.057 | -1.46 | 0.034 | 1.0001 | 0 | 0 | 0 | 0 | 0 |
| D1O36_04345 | XRE family transcriptional regulator | -2.00 | 0.007 | -1.41 | 0.125 | 1 | 0 | 0 | 0 | 0 | 0 |
| D1O36_04410 | hypothetical protein | -1.72 | 0.045 | -1.07 | 0.143 | 1 | 0 | 0 | 0 | 0 | 0 |
| D1O36_05305 | glycosyltransferase family 2 protein | -1.75 | 0.027 | -1.72 | 0.408 | 1.0001 | 0 | 0 | 0 | 0 | 0 |
| D1O36_05310 | IS6 family transposase | -1.49 | 0.021 | -1.79 | 0.319 | 1.0001 | 0 | 0 | 0 | 0 | 0 |
| D1O36_05315 | flippase | -1.33 | 0.043 | -1.82 | 0.263 | 1.0001 | 0 | 0 | 0 | 0 | 0 |
| D1O36_05350 | LytR family transcriptional regulator | -1.57 | 0.018 | -0.69 | 0.741 | 0.9882 | 0.0044 | 0.0001 | 0 | 0 | 0.0073 |
| D1O36_06210 | truncated hypothetical protein | -1.68 | 0.018 | -2.04 | 0.084 | 1 | 0 | 0 | 0 | 0 | 0 |
| D1O36_06360 | DUF5067 | -1.13 | 0.021 | -1.46 | 0.256 | 0 | 0 | 1.0001 | 0 | 0 | 0 |
| D1O36_06370 | hypothetical protein | -1.38 | 0.130 | -1.12 | 0.001 | 1 | 0 | 0 | 0 | 0 | 0 |
| D1O36_06830 | hypothetical protein | -0.95 | 0.043 | -0.33 | 0.787 | 1 | 0 | 0 | 0 | 0 | 0 |
| D1O36_06855 | truncated hypothetical protein | -2.04 | 0.005 | -2.51 | 0.039 | - | - | - | - | - | - |
| D1O36_07100 | GntR family trans regulator (YtrA) | -0.14 | 0.498 | 1.69 | 0.002 | 1.0001 | 0 | 0 | 0 | 0 | 0 |
| D1O36_07105 | ABC transporter ATP binding | 0.22 | 0.275 | 1.61 | 0.002 | 1 | 0 | 0 | 0 | 0 | 0 |
| D1O36_07325 | 30S ribosomal protein S21 | -1.20 | 0.095 | -2.56 | 0.031 | 1 | 0 | 0 | 0 | 0 | 0 |
| D1O36_07345 | amino acid ABC transporter substrate-binding protein | -0.87 | 0.018 | -0.24 | 0.790 | 0 | 0 | 1.0001 | 0 | 0 | 0 |
| D1O36_07590 | KH domain containing protein | -0.75 | 0.250 | -2.25 | 0.023 | 1.0001 | 0 | 0 | 0 | 0 | 0 |
| D1O36_07595 | 30S ribosomal protein S16 | -1.62 | 0.030 | -2.03 | 0.093 | 1 | 0 | 0 | 0 | 0 | 0 |
| D1O36_07795 | phosphoribosylanthanilate isomerase | 0.97 | 0.018 | 0.64 | 0.741 | 1.0001 | 0 | 0 | 0 | 0 | 0 |
| D1O36_08190 | DNA-binding protein | -0.94 | 0.198 | -1.40 | 0.023 | 1.0001 | 0 | 0 | 0 | 0 | 0 |
| D1O36_08245 | *blpX* | 0.59 | 0.051 | 1.43 | 0.011 | 1 | 0 | 0 | 0 | 0 | 0 |
| D1O36_08250 | *blpQ* | 0.99 | 0.000 | 1.57 | 0.001 | 1.0001 | 0 | 0 | 0 | 0 | 0 |
| D1O36_08255 | *blpG* | 5.01 | 0.000 | 5.74 | 0.000 | 0.0004 | 0.9988 | 0.0003 | 0.0002 | 0.0002 | 0.0001 |
| D1O36_08260 | ISSth1b family transposase | 1.60 | 0.000 | 2.52 | 0.000 | 1.0001 | 0 | 0 | 0 | 0 | 0 |
| D1O36_08265 | *blpK* | 2.74 | 0.000 | 5.36 | 0.000 | 0.53 | 0.4661 | 0.0017 | 0.001 | 0.0005 | 0.0006 |
| D1O36_08270 | ORF 4/5/6 | 1.14 | 0.000 | 2.35 | 0.000 | 1 | 0 | 0 | 0 | 0 | 0 |
| D1O36_08275 | ORF 3 | 1.28 | 0.000 | 2.45 | 0.000 | 1 | 0 | 0 | 0 | 0 | 0 |
| D1O36_08280 | *blpU* | 1.55 | 0.000 | 2.69 | 0.000 | 0.6665 | 0.3315 | 0.0009 | 0.0004 | 0.0003 | 0.0003 |
| D1O36_08285 | ORF 1/2 | 6.25 | 0.000 | 6.29 | 0.000 | 0.7499 | 0.248 | 0.0009 | 0.0004 | 0.0002 | 0.0005 |
| D1O36_08290 | *blpD* | 4.87 | 0.000 | 5.04 | 0.000 | 0.7961 | 0.1972 | 0.0038 | 0.0009 | 0.0007 | 0.0013 |
| D1O36_08305 | *blpC* | 4.34 | 0.000 | 4.94 | 0.000 | 0.9729 | 0.0256 | 0.0012 | 0.0001 | 0.0001 | 0.0001 |
| D1O36_08310 | *blpB* | 5.34 | 0.000 | 5.36 | 0.000 | 0.9992 | 0.0004 | 0 | 0 | 0 | 0.0004 |
| D1O36_08315 | *blpA* | 5.56 | 0.000 | 5.42 | 0.000 | 1 | 0 | 0 | 0 | 0 | 0 |
| D1O36_08950 | DUF3397 | -1.53 | 0.002 | -0.28 | 0.824 | 1 | 0 | 0 | 0 | 0 | 0 |
| D1O36_09350 | *cadA* | -0.90 | 0.043 | -0.03 | 0.975 | 1.0001 | 0 | 0 | 0 | 0 | 0 |
| D1O36_09380 | transcriptional regulator | -1.98 | 0.021 | -1.10 | 0.225 | 1.0001 | 0 | 0 | 0 | 0 | 0 |
| D1O36_09675 | XRE family transcriptional regulator | -1.98 | 0.033 | -0.94 | 0.485 | 0.9999 | 0 | 0 | 0 | 0 | 0 |
| D1O36_09695 | XRE family transcriptional regulator | -2.13 | 0.018 | -1.23 | 0.239 | 1 | 0 | 0 | 0 | 0 | 0 |
| D1O36_09730 | 50s ribosomal protein L28 | -0.89 | 0.246 | -2.79 | 0.031 | 1 | 0 | 0 | 0 | 0 | 0 |
| D1O36_09810 | rpmF | -1.49 | 0.016 | -1.25 | 0.408 | 1.0001 | 0 | 0 | 0 | 0 | 0 |
| D1O36_03435* | bacteriocin immunity protein* | -1.12 | 0.171 | -1.18 | 0.036 | 1 | 0 | 0 | 0 | 0 | 0 |
| D1O36_04115* | histidine kinase* | -1.60 | 0.027 | -0.99 | 0.590 | 0.7935 | 0.0012 | 0.0002 | 0 | 0 | 0.2051 |

**References**

Teufel, F., Almagro Armenteros, J. J., Johansen, A. R., Gislason, M. H., Pihl, S. I., Tsirigos, K. D., et al. (2022). SignalP 6.0 predicts all five types of signal peptides using protein language models. Nat Biotechnol, 40(7), 1023-1025. doi: 10.1038/s41587-021-01156-3
